# Standardized Minimum Number of Individuals (MNI) Enumeration and Veterinary Forensic Assessment in a Large-Scale Animal Starvation Scene: A Technical Note

**DOI:** 10.3390/ani16172807

**Published:** 2026-09-07

**Authors:** Ah-Young Kim, BokKyung Ku, Kyunghyun Lee

**Affiliations:** Animal Disease Diagnostic Division, Animal and Plant Quarantine Agency, Gimcheon 39660, Republic of Korea; mochsha@korea.kr (A.-Y.K.);

**Keywords:** mass mortality, minimum number of individuals, skull-based enumeration, body condition score, starvation, standardization

## Abstract

Mass companion animal mortality scenes pose major veterinary forensic and animal welfare challenges, particularly when remains are commingled, variably decomposed, or skeletonized. In March 2023, the remains of 1256 dogs and cats were recovered from a private residence in Yangpyeong-gun, Gyeonggi-do, Republic of Korea, after prolonged deprivation of food and water. Because whole-carcass enumeration was not feasible across the entire site, a standardized skull-based minimum number of individuals (MNI) approach was used to derive the scene-wide count by combining direct counts of intact, non-duplicated carcasses with counts of isolated skulls from disarticulated remains. A representative subset of 22 specimens, consisting of 16 carcasses submitted by the investigating police and 6 isolated skulls collected during the scene investigation, underwent detailed forensic examination including postmortem radiography, full necropsy, and toxicological screening. Body condition scoring was possible in 5 of the 16 carcasses, and all five scored 1 to 3 on a 9-point scale, consistent with severe to extreme emaciation. Toxicological screening of all 16 carcasses was negative for all tested substance classes. Gross and Radiographical examination of the 6 isolated skulls identified external traumatic fractures and scavenging punctures in 3 specimens, breed-typical persistent open fontanels without evidence of trauma in 2, and an indeterminate suture-associated lesion in 1. This report proposes a practical and reproducible veterinary forensic workflow for large-scale companion animal mortality scenes in which extensive decomposition prevents reliable direct enumeration. The combined use of skull-based MNI estimation, structured necropsy, whole-body postmortem radiography, toxicological exclusion testing, and standardized skull morphology assessment supports evidentiary documentation and courtroom prosecution in mass animal cruelty investigations.

## Simple Summary

In March 2023, the remains of 1256 dogs and cats were found at a private residence in Yangpyeong-gun, Gyeonggi-do, South Korea, after the animals had reportedly been deprived of food and water for about three years. Because the bodies were commingled and in various stages of decomposition, a whole-carcass count was not possible, so researchers developed a skull-based counting method, assuming each animal contributed one skull, to estimate the total number of individuals at the scene. A representative group of 22 specimens (16 carcasses and 6 isolated skulls) was examined in detail, showing severe emaciation, empty stomachs, and negative results on poison testing, supporting starvation as the cause of death. Examination of the isolated skulls also helped distinguish real trauma injuries from normal breed-related skull features, such as open fontanels seen in small dog breeds. This report presents a practical, standardized workflow combining skull-based counting, nutritional scoring, and toxicology testing that can be used in future veterinary forensic investigations of large-scale animal neglect and cruelty cases.

## 1. Introduction

Severe neglect resulting in mass companion animal mortality represents one of the most extreme forms of animal abuse because it combines prolonged, preventable suffering with substantial forensic complexity. In large-scale scenes, remains may be commingled, fragmented, or distributed across a broad range of postmortem conditions, making conventional whole-carcass counting unreliable and complicating individual-based interpretation [1]. Under such circumstances, veterinary forensic investigation must address two parallel questions that are central to both animal welfare law and evidentiary documentation: how many animals are represented, and what diagnostic findings support the cause of death and the broader forensic interpretation of the scene [1].

In March 2023, a large number of dead dogs and cats were discovered at a private residence in Yangpyeong-gun, Gyeonggi-do, rovince, Republic of Korea. The investigation indicated that the animals had been deprived of food and water for approximately three years. The recovered remains ranged from relatively preserved carcasses to advanced decomposition and complete skeletonization, creating substantial difficulty in establishing a defensible total number of individuals represented at the scene and in selecting specimens suitable for detailed forensic examination [2,3]. The perpetrator had systematically acquired surplus, sick, or abandoned animals from local breeding facilities and commercial brokers under the pretext of care, only to confine them without food and water until fatal starvation occurred [2,3].

Minimum number of individuals (MNI) estimation is a well-established concept in forensic anthropology and zooarchaeology for the interpretation of commingled skeletal assemblages [4]. However, practical veterinary applications of MNI estimation in mass companion animal mortality scenes have rarely been described in a standardized and reproducible manner [4,5]. In parallel, establishing starvation as a cause of death in suspected cruelty cases requires more than documentation of low body condition alone. It requires structured gross assessment, postmortem radiographic evaluation, exclusion of competing toxic causes, and careful interpretation of skeletal findings that might otherwise be overinterpreted as inflicted trauma.

The present report reframes this case as a standardization-oriented veterinary forensic protocol rather than as a single isolated case report. The aims of the study were (1) to describe a skull-based MNI workflow suitable for scene-wide enumeration in a mass companion animal mortality setting in which direct whole-carcass counting was not feasible; (2) to present a structured forensic assessment framework for a representative subset of remains, including nutritional, toxicological, virological, radiological, and skeletal evaluation; and (3) to demonstrate how these components can be integrated into a practical, court-defensible investigative approach that is transferable to future veterinary forensic casework [4,5].

## 2. Materials and Methods

### 2.1. Case Background and Specimen Selection

The investigation involved a private residence in Yangpyeong-gun, Gyeonggi Province, Republic of Korea, from which a total of 1256 animal remains were recovered. Among the 1256 recovered remains, dogs constituted the overwhelming majority (*n* = 1248, 99.36%), while cats comprised a minor proportion (*n* = 8, 0.64%), reflecting the suspect’s operational pattern of acquiring surplus or retired breeding canines. The remains represented a broad postmortem spectrum, ranging from recently deceased carcasses to partially disarticulated and fully skeletonized individuals. Because not all remains were suitable for complete necropsy, a representative subset of 22 specimens was selected for detailed forensic evaluation at the Animal and Plant Quarantine Agency (APQA).

The examined subset included 16 carcasses submitted by the investigating police station on the basis of legal relevance and tissue preservation, together with 6 isolated skulls collected separately during the on-site veterinary investigation because of notable morphologic features or suspected trauma. All 16 police-submitted carcasses were dogs; feline remains were recovered in states of advanced autolysis or skeletonization and were not prioritized by law enforcement for soft-tissue pathological or toxicological evaluation. All 16 carcasses underwent full-body postmortem radiography, gross necropsy, and toxicological testing. The 6 isolated skeletonized skulls underwent detailed anatomical and osteological evaluation.

### 2.2. Standardized Scene Investigation and Evidence Handling

**Figure 1:**
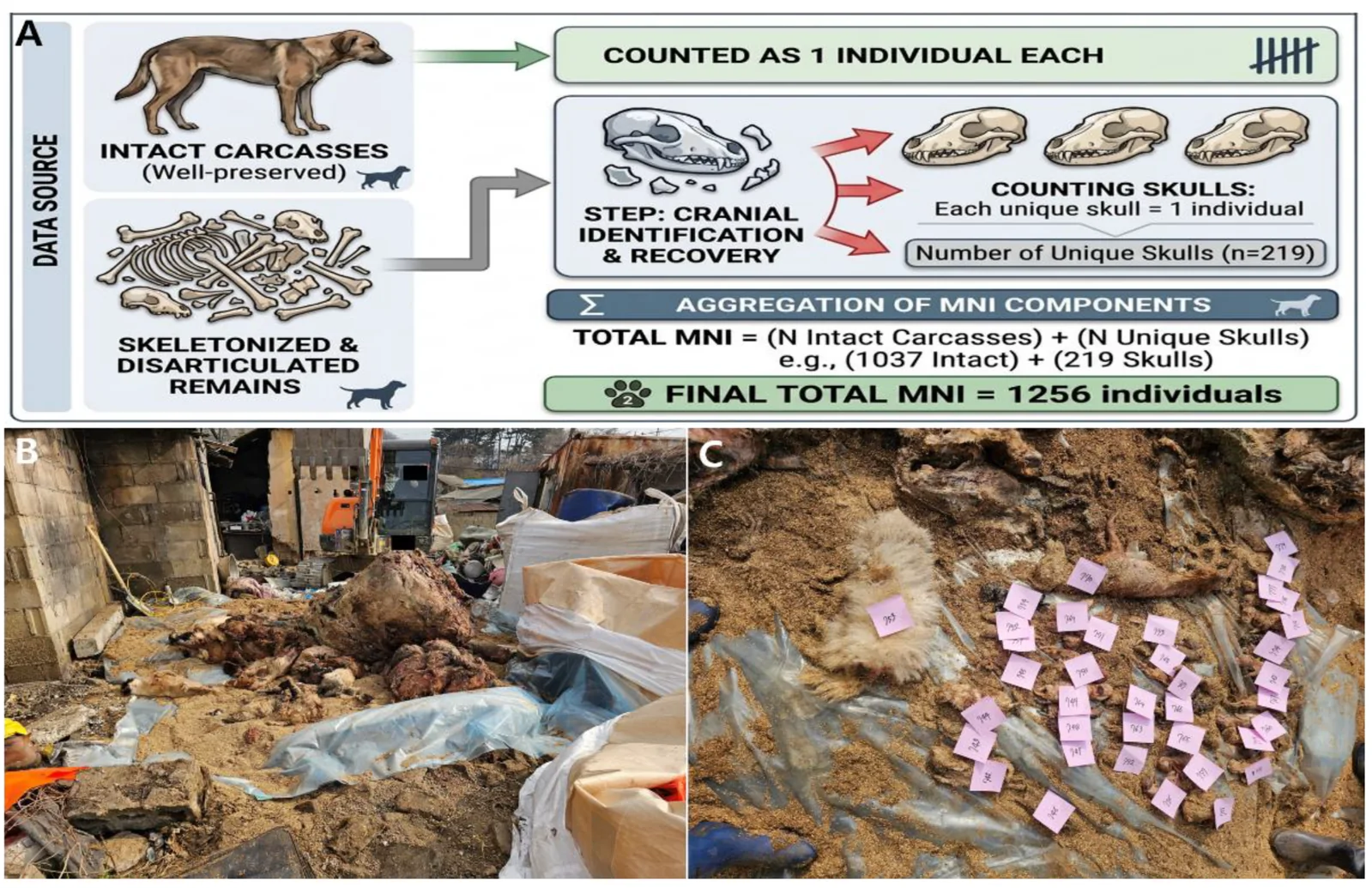
Comprehensive overview of the canine mass mortality event enumeration process. (**A**) Schematic overview of MNI estimation in a canine mass mortality event. (**B**) Partial view of the complex mass mortality scene. (**C**) On-site enumeration in progress, using numerical tags to mark individual remains for counting.

For standardization purposes, the scene investigation was conceptualized as a sequential workflow. First, remains were classified according to preservation status as intact carcasses, decomposed carcasses, disarticulated skeletal remains, or isolated skeletal elements. Here, ‘intact carcasses’ refers to structurally continuous, non-disarticulated anatomical units, irrespective of the internal degree of soft-tissue decomposition or putrefactive liquefaction. Each recoverable unit was documented and handled as a distinct evidentiary item. Where possible, photographic documentation and traceable specimen identification were maintained throughout collection, transport, and laboratory examination (Figure 1).

Second, remains judged to represent intact, non-duplicated individuals were counted directly. Commingled or skeletonized material that could not be reliably assigned to a complete carcass was routed to skeletal enumeration. Third, laboratory submission of selected specimens was guided by evidentiary significance, preservation status, and the presence of notable lesions or structural abnormalities. This workflow was designed to provide a conservative, auditable, and court-defensible framework for large-scale veterinary forensic investigation.

### 2.3. Skull-Based Minimum Number of Individuals Estimation

Because advanced decomposition and skeletonization precluded reliable direct enumeration of all remains, a skull-based MNI approach was used. The method was based on the premise that each individual animal contributes a single cranium. The final scene-wide total was therefore calculated by summing: (1) intact, non-duplicated carcasses identified as discrete individuals; and (2) additional skulls identified among disarticulated or skeletonized remains that were not already associated with a counted carcass.

This approach was intentionally conservative. In cases of uncertainty, counts were resolved in a manner that avoided overestimation. The resulting value was interpreted as the minimum number of individuals represented at the scene. The protocol acknowledges that the final count may underestimate the true number of animals if skulls were absent, destroyed, fragmented beyond recognition, or not recovered.

### 2.4. Nutritional Assessment and Postmortem Radiography

Nutritional status was evaluated using the validated 9-point body condition score (BCS) system [6], in which 1 indicates extreme emaciation, 4–5 indicates ideal body condition, and 9 indicates severe obesity. Gross visual and manual examination were used to assess the prominence of ribs, vertebrae, and pelvic bones, as well as depletion of subcutaneous, visceral, pericardial, and perirenal fat depots.

Gastrointestinal contents were also assessed when preserved sufficiently for interpretation. Because a proportion of the remains showed advanced autolysis or represented juvenile animals, BCS was recorded as not assessable where decomposition or developmental stage precluded reliable scoring.

Prior to necropsy dissection, postmortem whole-body radiography (digital radiography, DR; ventrodorsal and lateral views) was systematically performed on all 16 submitted carcasses. Radiographs were evaluated to assess skeletal integrity, detect occult fractures, identify radio-dense foreign bodies or metallic fragments (such as projectiles or microchips), and evaluate internal gas patterns associated with postmortem decomposition.

### 2.5. Toxicological and Virological Testing

To exclude chemical poisoning as a competing cause of death, stomach contents and visceral organs from the submitted carcasses were submitted for broad toxicological screening. The tested substance classes included cyanide, organophosphates, organochlorines, carbamates, rodenticides, barbiturates, benzodiazepines, phenothiazines, salicylates, and toxic alkaloids.

To assess possible infectious contributions or comorbidities, PCR assays were performed on pooled tissue samples, including brain, spleen, lung, heart, kidneys, liver, intestine, and feces, using in-house protocols routinely applied at APQA. The viral targets for canine cases included canine parvovirus (CPV), canine distemper virus (CDV), canine adenovirus (CAV), canine herpesvirus (CHV), canine coronavirus (CCoV), canine parainfluenza virus (CPIV), and canine influenza virus (CIV).

### 2.6. Skull Morphological and Osteological Assessment

The 6 isolated skulls were examined grossly and osteologically to characterize cranial defects and to distinguish traumatic lesions from natural anatomic variation and taphonomic artifacts. Lesions were assessed with attention to margin character, relationship to cranial sutures, distribution, beveling, and overall morphology.

Defects crossing expected sutural boundaries and showing irregular, non-sutural fracture morphology were interpreted as consistent with external mechanical trauma or compressive force. Smoothly margined, midline vault openings without associated secondary fracture features were interpreted as compatible with breed-typical open fontanels commonly seen in brachycephalic and toy breeds (e.g., Chihuahuas). Puncture defects with cortical indentation were assessed for postmortem animal scavenging. Defects that closely followed cranial sutures were classified as indeterminate when structural weakness along the suture could not be excluded.

## 3. Results

### 3.1. Scene-Wide Enumeration

Application of the skull-based protocol yielded a defensible scene-wide MNI of 1256 individual animals (Table 1). This total comprised 1248 dogs (99.36%) and 8 cats (0.64%). This value incorporated both direct counts of intact, non-duplicated carcasses (n = 1037, including 16 examined and 1021 simply counted) and cranial counts from disarticulated or skeletonized remains (*n* = 219, including 6 examined and 213 simply counted). A total of 22 specimens, representing 1.75% of the scene-wide population, were selected for detailed laboratory forensic examination (Table 1).

### 3.2. Nutritional, Radiographic, and Gross Pathologic Findings

**Figure 2:**
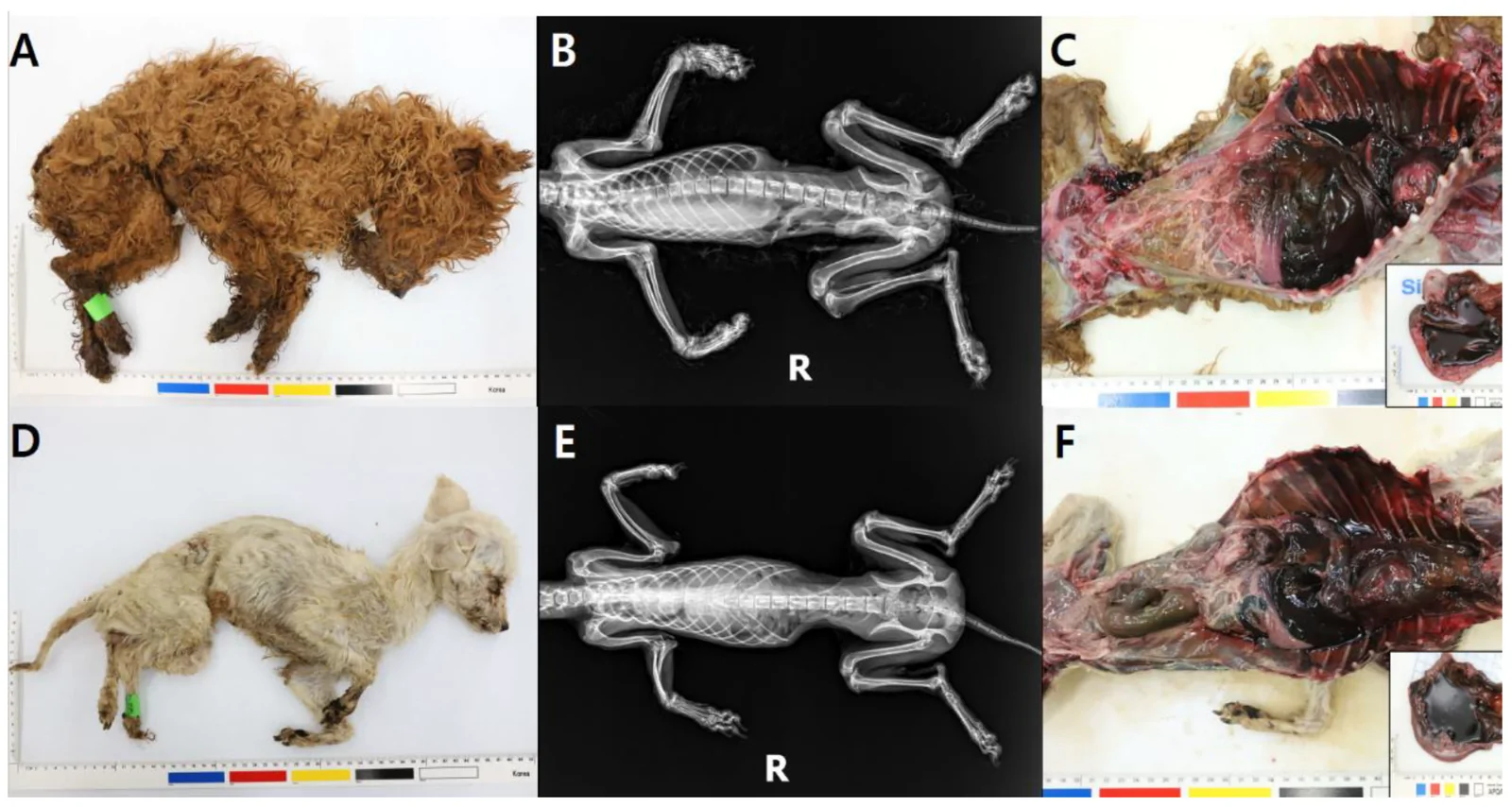
Key morphological and postmortem radiographic findings in BCS 1 individuals diagnosed with fatal starvation. (**A**) Lateral gross photograph of specimen ID 23Q051 showing severe emaciation and skeletal prominence. (**B**) Radiographic ventrodorsal projection of specimen ID 23Q051 demonstrating absence of traumatic fractures or radiopaque foreign bodies. R, right side. (**C**) Marked visceral fat depletion in specimen ID 23Q051, with (inset) empty gastric lumen lacking food contents. (**D**) Lateral gross photograph of specimen ID 23Q052. (**E**) Radiographic ventrodorsal projection of specimen ID 23Q052 showing skeletal integrity without traumatic disruption. R, right side. (**F**) Severe wasting of abdominal adipose tissue in specimen ID 23Q052, with (inset) empty stomach.

Whole-body postmortem radiography conducted on all 16 carcasses revealed no radiopaque projectile fragments, metallic foreign bodies, or acute traumatic skeletal fractures (Figure 2B,E). Radiographic findings were notable primarily for generalized soft-tissue volume reduction and postmortem decompositional gas accumulation.

Among the 16 police-referred carcasses, body condition scoring was possible in 5 animals, all of which scored between 1 and 3 on the 9-point scale. These findings indicated severe to extreme emaciation. In three dogs assigned a BCS of 1, including one pregnant female carrying six early-stage fetuses (23Q049) and one dog with concurrent chronic dental trauma (23Q054), subcutaneous, visceral, pericardial, and perirenal fat reserves were essentially depleted, and gastric contents were absent (Table 2, Figure 2).

BCS could not be assigned to the remaining 11 carcasses because of severe autolysis in 3 cases and juvenile age in 8 cases. In two carcasses (23Q042 and 23Q044), advanced putrefactive liquefaction and postmortem insect colonization prevented anatomical sex determination despite intact carcass continuity. Nevertheless, the assessable carcasses consistently showed a pattern compatible with prolonged nutritional deprivation.

### 3.3. Toxicological and Virological Findings

Toxicological screening of all 16 carcasses was negative for all tested substance classes. Virological PCR testing revealed positivity for CPV and CCoV in several juvenile and adult carcasses (Table 2), indicating endemic enteric viral exposure common in crowded breeding or holding environments. Together with the marked depletion of fat reserves, absence of significant gastric contents in severely emaciated animals, and negative toxicological findings, fatal exogenous starvation was supported as the primary cause of death (Table 2).

### 3.4. Skull Morphology and Osteological Findings

**Figure 3:**
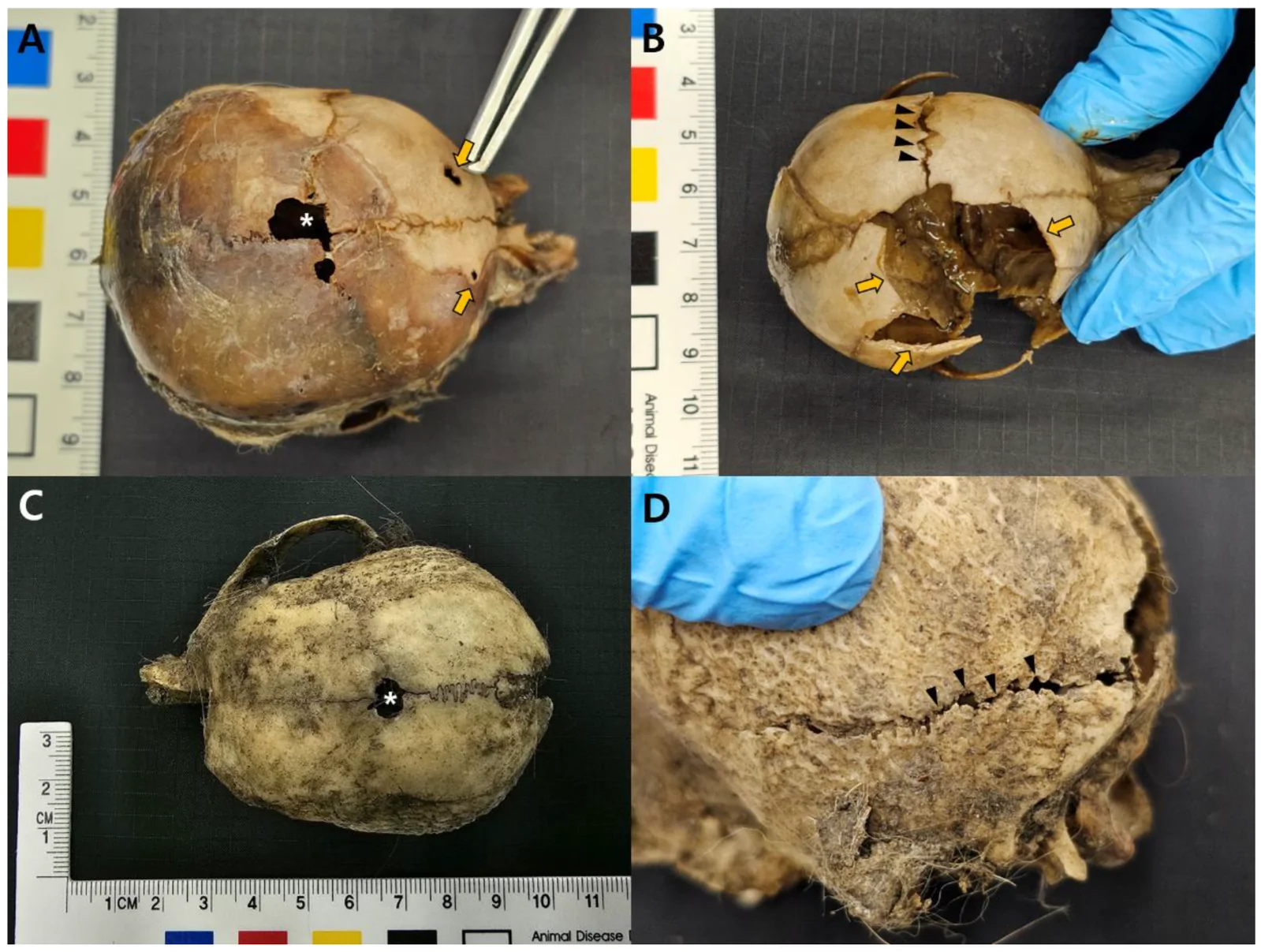
Gross and osteological findings in skeletonized skulls. (**A**) Perforations from puncture-type trauma (yellow arrows) presumed to result from animal scavenging, distinguished from the open fontanel (white asterisk). (**B**) Atypical non-sutural fracture lines (yellow arrows) existing independently of cranial sutures (black arrowheads), indicative of compressive or mechanical forces. (**C**) Persistent open fontanel (white asterisk), a breed-specific developmental finding observed in small/brachycephalic breeds without traumatic reaction. (**D**) Indeterminate cranial separation coinciding with a cranial suture (black arrowheads).

Gross and osteological examination of the 6 isolated skeletonized skulls identified three specimens with distinct cranial defects (Figure 3A–C). Specimen 1 exhibited focal circular perforations with cortical crushing (Figure 3A), consistent with postmortem carnivore scavenging punctures rather than vital inflicted trauma. Specimen 2 showed atypical non-sutural fractures crossing cranial plates (Figure 3B), consistent with mechanical compressive forces sustained either perimortem or postmortem during carcass compaction and burial. Two skulls showed smoothly margined midline vault openings typical of breed-associated persistent open fontanels without evidence of traumatic force (Figure 3C). One skull displayed a fracture line aligned directly with a cranial suture (Figure 3D) and was categorized as indeterminate. None of the cranial lesions presented osteogenic vital reaction, confirming unhealed or postmortem origin.

## 4. Discussion

The principal contribution of this report lies in the standardization of veterinary forensic workflow rather than in the unusual scale of the case alone. In mass companion animal mortality scenes, particularly those involving advanced decomposition or skeletonization, direct enumeration of complete bodies is often not possible [1]. Under such conditions, a skull-based MNI approach provides a practical and conservative alternative that can be incorporated into veterinary forensic casework with relative ease [4,5].

Using the cranium as the counting unit offers several operational advantages. The method is conceptually simple, relatively rapid, and easier to standardize than full osteological reconciliation based on multiple paired skeletal elements. Although the approach yields only a minimum estimate and may underestimate the true total when skulls are missing, destroyed, or not recovered, it nevertheless provides a defensible scene-wide value for evidentiary and investigative purposes.

A second strength of the present approach is the integration of scene-wide enumeration with structured examination of a representative evidentiary subset. In the examined carcasses, severe emaciation in assessable animals, marked depletion of multiple fat depots, absence of meaningful gastric contents, absence of radiographic fractures, and uniformly negative toxicological findings together supported fatal exogenous starvation as the primary cause of death in the examined subset [6,7]. This interpretation was also consistent with the case history of prolonged deprivation of food and water and illustrates the forensic value of combining gross pathological findings with exclusionary diagnostic testing in suspected neglect and cruelty investigations.

The skull morphology component of the protocol also has broader methodological importance. In small-breed dogs, breed-typical persistent open fontanels, suture-associated defects, and postmortem alterations may mimic traumatic lesions if interpretation is based solely on the presence of a cranial opening or fracture line. The use of explicit gross morphologic criteria therefore improves interpretive consistency and reduces the risk of false-positive conclusions regarding inflicted trauma in skeletonized remains. Furthermore, discriminating taphonomic artifacts (e.g., scavenging tooth marks or postmortem soil compaction fractures) from deliberate perimortem blunt force trauma is paramount in establishing an accurate forensic reconstruction.

In the subsequent judicial proceedings, the comprehensive veterinary forensic report—incorporating the standardized skull-based MNI quantification of 1256 animals, the pathological proof of extreme emaciation, and the systematic toxicological exclusion of acute poisons—served as pivotal prosecution evidence. The district court accepted the veterinary forensic findings in their entirety, establishing deliberate mass starvation under the South Korean Animal Protection Act and sentencing the defendant to the statutory maximum penalty of three years’ imprisonment, a landmark verdict subsequently affirmed by the appellate court [2,3]. This outcome highlights the direct legal efficacy and necessity of auditable forensic quantification in animal cruelty jurisprudence.

The present findings also highlight an important practical limitation of large-scale forensic investigations: only a small proportion of remains may be suitable for complete pathological examination [4,5]. Accordingly, the pathological and toxicological findings presented here should be interpreted as corroborative forensic evidence derived from a representative subset rather than as a statistical description of all 1256 animals represented at the scene. Even so, this combination of conservative enumeration and targeted forensic examination provides a useful framework for documenting large-scale animal cruelty cases in a manner that is reproducible, auditable, and evidentially robust.

## 5. Limitations

The skull-based method provides a minimum, not absolute, count of individuals. The total may be underestimated if skulls are missing, destroyed, or not recovered from the scene. In addition, because a fully reconciled postcranial MNI analysis was not performed, the count could not be cross-validated using paired limb elements or other osteological methods.

The forensic pathology and toxicology results were derived from only 22 of the 1256 animals represented at the scene. Accordingly, those findings should not be extrapolated statistically to the entire assemblage. Rather, they serve as targeted forensic evidence supporting the interpretation of starvation in the examined subset.

## 6. Conclusions

In conclusion, this technical note addresses the complex forensic and animal welfare challenges of mass companion animal neglect by fulfilling three primary objectives:

(1) Standardized Enumeration Workflow: A skull-based MNI approach provided a rapid, conservative, and defensible enumeration workflow, establishing a scene-wide minimum count of 1256 individual companion animals (1248 dogs and 8 cats) where severe commingling and advanced decomposition precluded conventional whole-body counting.

(2) Integrated Forensic Assessment Framework: Application of a structured diagnostic framework—combining body condition scoring, whole-body postmortem radiography, exclusionary toxicological testing, and differential cranial osteology—substantiated extreme emaciation (BCS 1–3) and fatal exogenous starvation while reliably distinguishing taphonomic scavenging defects and breed-typical open fontanels from inflicted mechanical trauma.

(3) Evidentiary Rigor and Legal Defensibility: The forensic documentation provided conclusive, court-defensible evidence that resulted in the defendant’s conviction and imposition of the statutory maximum three-year prison sentence under the Animal Protection Act. This framework offers a transferable and auditable standard for future veterinary forensic investigations involving large-scale animal cruelty and mass mortality casework.

## Figures and Tables

**Table 1:** Scene-wide enumeration and characteristics of specimens recovered from the mass mortality scene.

	Examined Cases			Simply Counted Cases			Total
	Whole Carcass	Skull Only	Subtotal	Whole Carcass	Skull Only	Subtotal	
Number	16	6	22	1021	213	1234	1256
Proportion (%)	1.27	0.48	1.75	81.29	16.96	98.25	100
Decomposition stage (No. of cases)	Bloat (1); Active decay (9); Advanced decay (6)	Skeletonization (6)	-	Active decay to Advanced decay (1021)	Skeletonization (213)	-	-
Type of examination (No. of cases)	Full necropsy (16); Radiography (16); Histopathology (10); Virology (10); Toxicology (2)	Anatomical & osteological examination (6)	-	N.A.	N.A.	-	-

**Table 2:** Necropsy, radiographic, virological, and toxicological results of the 16 police-referred dog carcasses.

Specimen ID	Breed	Age Group	Sex	BCS *	Decomp. **	Radiography (R)/Gross Findings	Virology ***	Toxicology §	Diagnosis ¶
23Q042	Miniature Poodle	Adult	Female	N.A.	Advanced decay	R: No fractures. Gross: No significant findings	N.A.	N.A.	Undetermined
23Q043	Miniature Poodle	Adult	Male	N.A.	Advanced decay	R: No fractures. Gross: No significant findings	N.A.	N.A.	Undetermined
23Q044	Unidentifiable	Adult	Female	N.A.	Advanced decay	R: No fractures. Gross: No significant findings	N.A.	N.A.	Undetermined
23Q045	Mixed	Juvenile	Female	N.A.	Active decay	R: No fractures. Gross: No gastric contents	CPV+, CCoV+	N.A.	Undetermined
23Q046	Mixed	Juvenile	Male	N.A.	Active decay	R: No fractures. Gross: No gastric contents	CPV+, CCoV+	N.A.	Undetermined
23Q047	Mixed	Juvenile	Female	N.A.	Active decay	R: No fractures. Gross: No gastric contents	CPV+, CCoV+	N.A.	Undetermined
23Q048	Mixed	Juvenile	Female	N.A.	Active decay	R: No fractures. Gross: No gastric contents	CPV+, CCoV+	N.A.	Undetermined
23Q049	Chihuahua	Adult	Female	2	Active decay	R: No fractures; early fetuses. Gross: 6 embryos; empty stomach	CPV+, CCoV+	N.A.	Starvation
23Q050	Mixed	Juvenile	Male	N.A.	Active decay	R: No fractures. Gross: No gastric contents; swollen axillary LN	CPV+, CCoV+	N.A.	Undetermined
23Q051	Miniature Poodle	Adult	Male	1	Active decay	R: No fractures; severe emaciation. Gross: Fat depletion; empty stomach	CPV+	N.A.	Starvation
23Q052	Mixed	Adult	Female	1	Active decay	R: No fractures; severe emaciation. Gross: Fat depletion; empty stomach	CPV+, CCoV+	N.A.	Starvation
23Q053	Mixed	Adult	Female	3	Active decay	R: No fractures. Gross: Bloody liquid in esophagus; subinvolution of uterus	CPV+	Not detected	Starvation
23Q054	Papillon	Adult	Female	1	Bloat	R: No acute fractures. Gross: Digested food; fat depletion; sub-conjunctival hem.; tooth fractures	CPV+	Not detected	Starvation
23Q055	Shiba Inu	Juvenile	Male	N.A.	Advanced decay	R: No fractures. Gross: No significant findings	N.A.	N.A.	Undetermined
23Q056	Mixed	Juvenile	Male	N.A.	Advanced decay	R: No fractures. Gross: No significant findings	N.A.	N.A.	Undetermined
23Q057	Mixed	Juvenile	Female	N.A.	Advanced decay	R: No fractures. Gross: No significant findings	N.A.	N.A.	Undetermined

* Body condition score (BCS) is rated on a 1 (emaciated) to 9 (highly obese) scale, with 4–5 considered ideal; BCS was not assessable (N.A.) for juveniles or specimens exhibiting advanced decay. ** Decomposition evaluated in five stages: fresh, bloat, active decay, advanced decay, skeletonization. *** Virological PCR panel includes canine parvovirus (CPV), canine distemper virus (CDV), canine adenovirus (CAV), canine herpesvirus (CHV), canine coronavirus (CCoV), canine parainfluenza virus (CPIV), and canine influenza virus (CIV). § Toxicological screening panel includes cyanides, organophosphates, organochlorines, carbamates, rodenticides, barbiturate derivatives, benzodiazepine derivatives, phenothiazine derivatives, salicylic acid derivatives, and other toxic alkaloids. ¶ Histopathological analysis was performed on select tissues, but postmortem autolysis severely limited diagnostic utility.

## Data Availability

Data are contained within the article. Additional case-linked materials may be available from the corresponding author on reasonable request, subject to legal, privacy, and evidentiary restrictions.
